# AutoPVPrimer: A comprehensive AI-Enhanced pipeline for efficient plant virus primer design and assessment

**DOI:** 10.1371/journal.pone.0317918

**Published:** 2025-01-30

**Authors:** Abozar Ghorbani, Mahsa Rostami, Elham Ashrafi-Dehkordi, Pietro Hiram Guzzi

**Affiliations:** 1 Nuclear Science and Technology Research Institute (NSTRI), Nuclear Agriculture Research School, Karaj, Iran; 2 Department of Food Hygiene and Quality Control, Nutrition Research Center, School of Nutrition and Food Sciences, Shiraz University of Medical Sciences, Shiraz, Iran; 3 Department of Surgical and Medical Sciences, Magna Graecia University of Catanzaro, Catanzaro, Italy; Indian Agricultural Research Institute, INDIA

## Abstract

Plant viruses pose a significant threat to global agriculture and require efficient tools for their timely detection. We present AutoPVPrimer, an innovative pipeline that integrates artificial intelligence (AI) and machine learning to accelerate the development of plant virus primers. The pipeline uses Biopython to automatically retrieve different genomic sequences from the NCBI database to increase the robustness of the subsequent primer design. The design_primers_with_tuning module uses a random forest classifier that optimizes parameters and provides flexibility for different experimental conditions. Quality control measures, including the evaluation of poly-X content and melting temperature, increase primer reliability. Unique to AutoPVPrimer is the visualize_primer_dimer module, which supports the visual evaluation of primer dimers—a feature missing in other tools. Primer specificity is validated via primer BLAST, which contributes to the overall efficiency of the pipeline. AutoPVPrimer has been successfully applied to the tomato mosaic virus, proving its adaptability and efficiency. The modular design allows customization by the user and extends the applicability to different plant viruses and experimental scenarios. The pipeline represents a significant advance in primer design and provides researchers with an effective tool to accelerate molecular biology experiments. Future developments aim to extend compatibility and incorporate user feedback to consolidate AutoPVPrimer as an innovative contribution to the bioinformatics toolbox and a promising resource for the advancement of plant virology research.

## 1. Introduction

Plant viruses pose significant threats to global agriculture, causing substantial economic losses by impacting crop yield and quality estimated at $30 billion worldwide each year [1]. They are a threat to global food security and sustainability and are responsible for nearly half of all emerging plant diseases worldwide [2]. The International Committee on Taxonomy of Viruses (ICTV) has recognized 14,690 virus species as of 2024, reflecting substantial growth from previous years. Plant viruses are classified based on their genomic structure, similar to animal viruses, and recent taxonomy updates have expanded the ranks of virus families, genera, and species. Research continues to prioritize viruses that infect economically significant crops, given their impact on agriculture [3]. A roster of common plant viruses demands attention due to their widespread prevalence and significant impact on global agriculture. Notable among them is the Tobacco Mosaic Virus (TMV), infamous for its devastating effects on the tobacco industry. Tomato Mosaic Virus (ToMV), a closely related tobamovirus, was selected for this study due to its well-documented genomic structure and significant impact on tomato production worldwide. Its inclusion in this research highlights its utility as a model virus for designing molecular tools to diagnose and manage plant viruses. Also, Tomato Yellow Leaf Curl Virus (TYLCV) poses a major threat to the tomato supply chain with its severe diseases. Tomato Spotted Wilt Virus (TSWV) wreaks economic havoc on various crops, while Cucumber Mosaic Virus (CMV) poses a pervasive threat to diverse crops. Potato Virus Y (PVY) significantly impacts global potato production, and the Cauliflower Mosaic Virus (CaMV) affects cruciferous vegetable production. African Cassava Mosaic Virus (ACMV) threatens cassava, Plum Pox Virus (PPV) affects stone fruit trees, Brome Mosaic Virus (BMV) impacts grain crops, and Potato Virus X (PVX) contributes to challenges in potato farming. Understanding the impact of these viruses is critical to developing effective strategies to mitigate their detrimental effects on agricultural productivity and global food security [4]. Navigating the intricate landscape of common plant viruses emphasizes the crucial need for effective molecular tools. Paramount among these tools is the precision of primer design, pivotal for nucleic acid-based diagnostics like PCR.

To address the challenges posed by various plant viruses, the development of robust molecular tools for their detection and characterization is imperative. Molecular tools commonly used include enzyme-linked immunosorbent assay (ELISA), polymerase chain reaction (PCR), RT-PCR, recombinase polymerase amplification (RPA) and loop-mediated isothermal amplification (LAMP), or a combination of these methods. Among these, traditional molecular techniques, such as PCR, have long been the cornerstone of nucleic acid-based diagnostics and are preferred due to their sensitivity, specificity, and ease of obtaining the required. However, the success of PCR critically depends on the design and specificity of primers used for target amplification. A specific fragment of DNA unique to the target pathogen is amplified through good primer design [5].

Primer design for plant viruses presents unique challenges due to the genomic variability among viral strains and the need for broad-spectrum primers that can target multiple variants. Conventional primer design methods often rely on manual curation, making them time-consuming and susceptible to biases introduced by individual researchers [6]. Moreover, the increasing volume of genomic data available demands innovative solutions to navigate the complexities of primer design for diverse and evolving viral populations.

Several factors contribute to the complexity of primer design, including the need for conserved regions to ensure broad specificity, optimal primer length, GC content, and melting temperature. Additionally, the potential formation of primer dimers (self and cross dimers), hairpins and off-target amplification poses challenges to the reliability of PCR assays [7]. Addressing these challenges requires a comprehensive and automated approach that integrates bioinformatics tools, machine learning, and quality control measures. The burgeoning field of bioinformatics and artificial intelligence (AI) provides unique opportunities to revolutionize primer design, offering more efficient and accurate approaches to combat plant viral diseases.

To address the challenges posed by various plant viruses, this study aims to develop robust molecular tools for their detection and characterization through the utilization of artificial intelligence and bioinformatics. The study focuses on the development of AutoPVPrimer, an innovative pipeline designed for the automated and optimized design of primers targeting plant viruses, to improve efficiency and precision in primer design methodologies. Unlike conventional methods, AutoPVPrimer offers unparalleled versatility and user-friendly adaptability, allowing researchers to customize parameters with ease. The standout feature is the design_primers_with_tuning module, which harnesses the power of a Random Forest Classifier, optimizing primer design parameters and significantly enhancing the probability of experimental success. This machine learning-driven approach is a paradigm shift, promising not only efficiency but also an unprecedented level of precision in primer design. Additionally, AutoPVPrimer introduces the visualize_primer_dimer module, a unique tool absent in other existing methods. This module provides researchers with a visual assessment of primer dimers, enhancing the interpretability of primer characteristics and ensuring the quality of the designed primers. AutoPVPrimer’s commitment to user-friendly innovation, integration of cutting-edge technologies, and emphasis on quality control measures positions it as a transformative and significant advancement in the field of molecular tools for plant virus diagnostics.

## 2. Materials and methods

### 2.1 Implementation details

**Fig 1:**
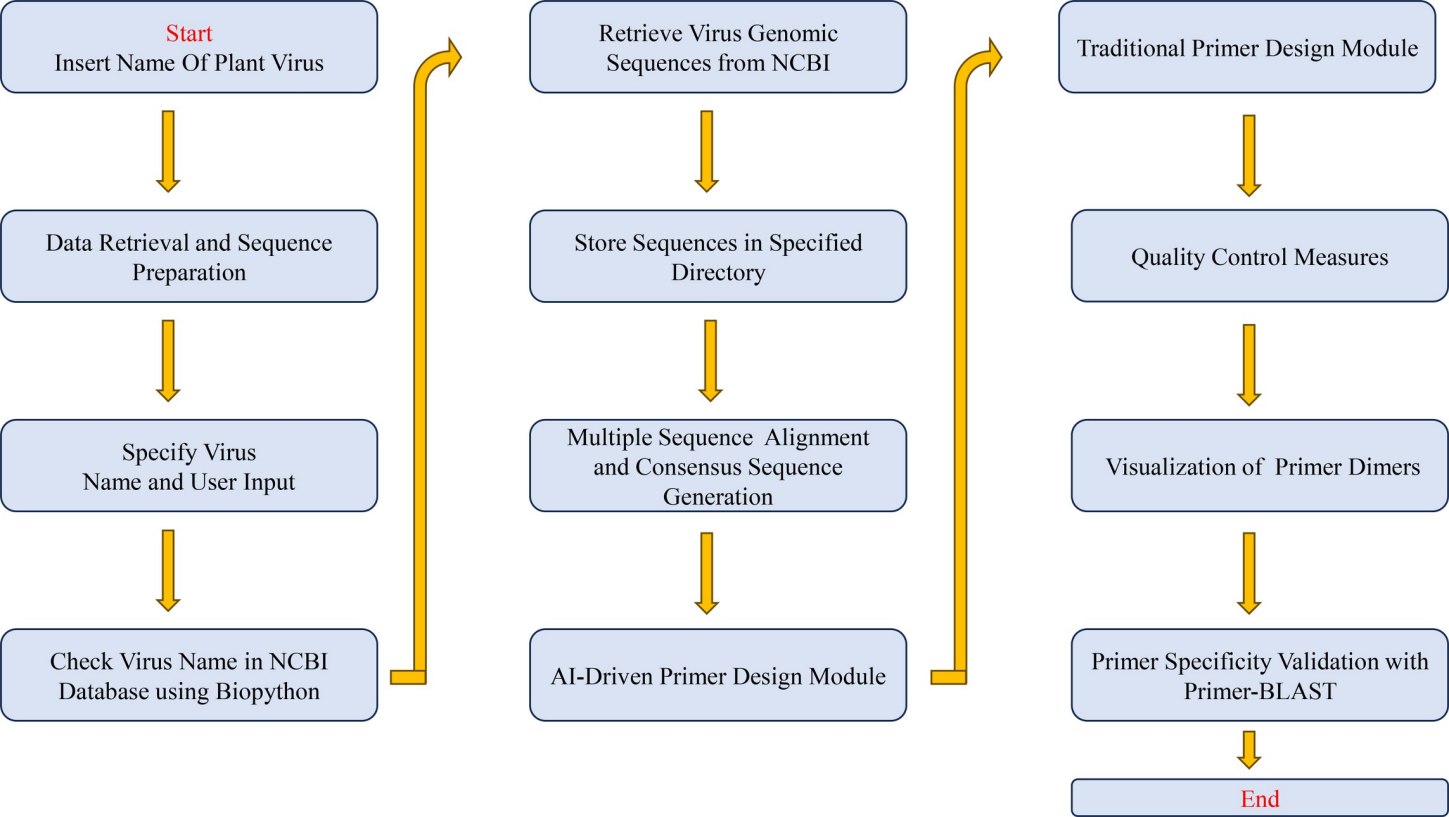
The Flowchart AutoPVPrimer shows how to design primers for plant viruses using AI-driven methods.

The flowchart illustrates (Fig 1) the AutoPVPrimer pipeline, implemented in Python 3 and leveraging key libraries such as Biopython, primer3-py, scikit-learn, and the Bio-Blast suite. Modularized for flexibility and maintainability, the pipeline comprises distinct sections: Data Retrieval and Sequence Preparation, Multiple Sequence Alignment and Consensus Sequence Generation, Primer Design and Optimization, Quality Control Measures, Visualization of Primer Dimers, and Primer Specificity Validation with Primer-BLAST. The modular design enables seamless integration with established bioinformatics tools, such as genome annotation pipelines, phylogenetic analysis frameworks, and high-throughput sequencing platforms. For example, AutoPVPrimer leverages Clustal Omega for sequence alignment and Primer-BLAST for specificity validation, facilitating interoperability with commonly used bioinformatics workflows. The pipeline begins with the automated retrieval of plant virus genome sequences from the NCBI and then goes through alignment, primer design, and quality control. At the same time, it allows researchers to upload their viral genomes for analysis, providing a streamlined and customizable approach to primer design for different virus populations. The unique module, visualize_primer_dimer, enhances interpretability by providing a visual assessment of primer characteristics, a notable feature absent in existing methods. The workflow’s adaptability, coupled with machine learning-driven optimization, positions AutoPVPrimer as an innovative and user-friendly tool for efficient primer design and evaluation.

### 2.2 Data retrieval and sequence preparation

The initial step of our pipeline involves the automatic retrieval of plant virus genomic sequences from the National Center for Biotechnology Information (NCBI) database (https://www.ncbi.nlm.nih.gov/). This is achieved through the implementation of the download_sequences.py script, which utilizes the Biopython library. Users input the specific plant virus name, and the script constructs a search query using the organism name and the term "genome." The script retrieves up to 10 Refseq genomes for the specified virus, and the sequences are stored in the specified output directory in FASTA format. To maintain data integrity, the script checks for the existence of the specified virus name in the NCBI database and provides user feedback in case of discrepancies. The use of the user’s email address in the Entrez module ensures compliance with NCBI’s usage policies.

### 2.3 Multiple sequence alignment and consensus sequence generation

The obtained virus genomic sequences are subjected to Multiple Sequence Alignment (MSA) using the create_alignment_and_contigs module. This step is essential for identifying conserved regions among the complete sequences, allowing for the creation of a consensus sequence that represents the shared genetic information of the targeted plant virus. The module uses the Biopython library to read the downloaded sequences, perform MSA with the Clustal Omega algorithm (Daugelaite et al., 2013), and generate a consensus sequence based on the majority nucleotide at each position. The resulting consensus sequence is saved in FASTA format for further analysis.

### 2.4 Consensus sequence algorithm

Given a set of aligned sequences of equal length, a consensus sequence is derived by examining each column in the multiple sequence alignment independently. The process typically follows these steps:

Initialization: Let *n* denote the total number of aligned sequences, and *L* represents the length of the alignment. The consensus sequence *C* is initialized as an empty string of length *L*.

Column Analysis: For each column *j* (where *j* ranges from 1 to *L*), create a frequency map *Fj* that counts the occurrence of each nucleotide ({A, C, T, G, -}) at that position across all *n* sequences.

Finding the Most Frequent Nucleotide: For each column *j*, identify the nucleotide *Nj* (from the set {A, C, G, T, -}) with the highest frequency in the frequency map *Fj*. If *Nj* is unique, append *Nj* to the consensus sequence *C*.

Tie Breaking: In the event of a tie, where two or more nucleotides share the highest frequency in the frequency map *Fj*, a pre-defined rule is applied to select the nucleotide to be appended to *C*. This rule can be as simple as the alphabetical order of nucleotides, where (A < C < G < T), or could be based on other prioritization mechanisms.

Consensus Assembly: Repeat steps 2 to 4 for each column *j* of the alignment. Concatenate all selected nucleotides *Nj* to construct the consensus sequence *C*.

Result: The final output *C* is the consensus sequence that represents the most commonly occurring nucleotide at each position of the alignment.

To codify this process into a mathematical formula, we could express the selection of the consensus nucleotide for each column *j* as:

[*Nj* = \arg\max_{*N* \in {A, C, G, T, -}} (*Fj* (*N*)) ]

Where (\arg\max) is the argument of the maximum, representing the nucleotide *N* that maximizes the frequency *Fj(N)* for column *j*. If this does not yield a unique nucleotide due to a tie, apply the tie-breaking rule.

### 2.5 Primer design and optimization

The core of the AutoPVPrimer pipeline lies in the primer design modules, which utilize traditional primer3 (Rozen and Skaletsky, 2000) alongside a novel machine learning-driven approach. This module is designed to offer flexibility to users by allowing them to specify parameters such as the number of primers to generate, product size range, and melting temperature range. For each iteration, the module defines a target region within the consensus sequence, randomly selecting a product size within the specified range. Primer3 is then employed to design primers for the target region, considering parameters such as optimal primer length, GC content, and melting temperature. The module repeats this process to generate the desired number of primer pairs.

In a novel approach, the design_primers_with_tuning module integrates machine learning to optimize primer design parameters. Features extracted from the designed primer pairs, including primer length and product size, serve as input for a Random Forest Classifier (RFC). The RFC was chosen for its robustness, ease of interpretation, and ability to handle high-dimensional data, which are essential for optimizing diverse primer properties effectively. Compared to more computationally intensive algorithms like XGBoost and LightGBM, RFC provides a computationally efficient solution suitable for researchers with varying computational resources. Randomized SearchCV is employed to identify the optimal parameters for the classifier, thereby enhancing the success rate of the designed primers.

This optimization approach ensures the efficient and precise design of primers for plant viruses, further enhancing the functionality and usability of the AutoPVPrimer pipeline. The optimization procedures described in the consecutive subsections build upon this foundation, creating a seamless and comprehensive primer design and evaluation process.

### 2.6 Quality control measures

To ensure the quality of the designed primers, a series of stringent quality control measures are implemented within the AutoPVPrimer pipeline. The check_primer_properties module performs an initial evaluation of primer characteristics, including the presence of poly-X regions, GC percentage, and differences in melting temperatures between forward and reverse primers. For instance, primers with a GC content outside the optimal range of 40–60% or with a Tm difference exceeding 5°C are automatically excluded from further analysis (Dieffenbach et al., 1993). This step serves as a preliminary filter to exclude primer pairs with significant deviations from optimal parameters, ensuring only baseline acceptable primers proceed further. For example, in a test dataset targeting ToMV, 15% of primer candidates were rejected at this stage due to excessive GC content, while 10% were excluded for poly-X regions exceeding four consecutive bases. By systematically filtering suboptimal primers, the module significantly reduces the likelihood of amplification issues in downstream applications, ensuring that only high-quality primers are considered for further evaluation and experimental validation.

Following the initial assessment of primer properties, additional quality control steps are conducted to further enhance the reliability of the designed primers. The check_primer_dimers module evaluates the potential formation of self and cross-dimers, as well as hairpin structures, by calculating the free energy and melting temperature using primer3-py. Any primer pairs that exhibit unfavorable dimerization tendencies are flagged for review and potential exclusion.

Moreover, the check_primer_quality module conducts a detailed thermodynamic analysis of primer characteristics, including primer length, GC content, and melting temperature, while also calculating enthalpy (ΔH), entropy (ΔS), and free energy (ΔG). This comprehensive evaluation ensures that the primers meet stringent quality standards and are suitable for reliable PCR amplification. By utilizing established formulas for calculating thermodynamic properties, including the enthalpy change and entropy change during the melting process, the module provides a comprehensive evaluation of each primer pair’s quality and suitability for experimental use.

[\Delta G = \Delta H—T \Delta S ]

Where:

- ΔH is the enthalpy change during the process
- T is the temperature in Kelvin
- ΔS is the entropy change during the process

[Tm = \frac{\Delta H}{\Delta S + R \ln(\frac{c}{4})} ]

Where:

- ΔH is the enthalpy change associated with the melting process
- ΔS is the entropy change associated with the melting process
- R is the ideal gas law constant
- c is the molarity of the monovalent cations

### 2.7 Visualization of primer dimers

To aid researchers in the visual assessment of primer dimers, the visualize_primer_dimer module plots melting curves for both forward and reverse primers. This graphical representation allows for a quick evaluation of the primer pairs’ suitability for experimental applications.

### 2.8 Primer specificity validation with Primer-BLAST

The designed primers undergo a specificity validation step using Primer-BLAST (Ye et al., 2012) through the run_primer_blast module. This step ensures that the primers do not exhibit significant homology with unintended targets in the NCBI nucleotide database. For each primer pair, the forward and reverse primers are concatenated into a single sequence, and Primer-BLAST is executed with specified parameters, including the nucleotide database, word size, and expected value. The results are saved in XML format, providing detailed information on the specificity of the designed primers.

### 2.9 Integration of machine learning for primer design optimization

The machine learning-driven approach within AutoPVPrimer is a cornerstone of its innovative primer design methodology. Specifically implemented in the design_primers_with_tuning module, this approach involves a Random Forest Classifier to optimize key primer design parameters. As the module generates primer pairs, features extracted from these pairs, such as primer length and product size, serve as inputs for the classifier. The Random Forest Classifier, facilitated by Randomized SearchCV, identifies optimal parameters for primer design, thereby significantly enhancing the success rate of the designed primers. To ensure transparency, AutoPVPrimer calculates feature importance metrics, which rank the influence of each parameter (e.g., primer length, GC content) on the classifier’s predictions. These metrics provide users with insights into the model’s decision-making process, promoting trust and enabling informed adjustments. This novel integration of machine learning ensures not only efficiency but also an unprecedented level of precision in primer design for plant viruses. The detailed workings of the machine learning model, its training data, and the interplay of features during parameter optimization contribute to the overall sophistication of AutoPVPrimer.

The primer design and optimization module in AutoPVPrimer empowers users with a flexible and customizable approach to fine-tune primer design parameters, ensuring adaptability to diverse experimental needs. Users can specifically tailor the following parameters:

1. Number of Primers: Users have the flexibility to define the desired quantity of primer pairs for a designated target region.
2. Product Size Range: AutoPVPrimer enables users to set the acceptable range for product sizes, granting control over the amplified DNA fragment sizes.
3. Melting Temperature Range: Users can customize the range of melting temperatures for the designed primers, ensuring alignment with their experimental conditions.

The innovative aspect of AutoPVPrimer lies in the integration of the Random Forest Classifier within the design_primers_with_tuning module. While the specific hyperparameters of the Random Forest Classifier, such as the number of trees in the forest, maximum tree depth, and minimum samples per leaf, are not initially exposed to maintain simplicity, advanced users have the option to fine-tune them for further model optimization. This user-centric customization not only harnesses the efficiency of automated primer design but also allows fine-tuning of the underlying machine learning model, delivering a tailored and optimized approach to plant virus primer design.

### 2.10 Ethical consideration

In addition to its robust primer design capabilities, AutoPVPrimer prioritizes user privacy and adheres to ethical considerations in line with data protection norms. The user-friendly interface necessitates input of the user’s email address for the retrieval of plant virus genomic sequences from the NCBI database. This measure ensures compliance with NCBI’s usage policies, contributing to responsible data utilization. AutoPVPrimer incorporates stringent data privacy measures to safeguard user information, reflecting our commitment to ethical practices in bioinformatics research. We recognize the importance of maintaining user trust and confidentiality in handling sensitive data, and our pipeline reflects these principles throughout the primer design process.

## 3. Results

### 3.1 Data retrieval and sequence preparation

The code was submitted to GitHub (https://github.com/Abozarghorbani/AutoPVPrimer.git). The download_sequences.py script successfully retrieved genomic sequences for "Tomato Mosaic Virus" from the NCBI nucleotide database. A total of 10 sequences were downloaded and saved in the specified output directory (/home/Your path) This step ensures a diverse representation of the virus’s genetic variability, contributing to the robustness of subsequent primer design. The utilization of the Biopython library for sequence retrieval and storage enhances the script’s efficiency and reliability. The integration of the user’s email address aligns with NCBI’s guidelines and supports the responsible use of their resources. The successful retrieval of diverse sequences from the NCBI nucleotide database for "Tomato Mosaic Virus" is a key success. This achievement ensures a robust representation of the virus’s genetic variability, contributing to the effectiveness of subsequent primer design.

### 3.2 Multiple sequence alignment and consensus sequence generation

The create_alignment_and_contigs module effectively performed MSA on the retrieved ToMV sequences. The aligned sequences were then used to generate a consensus sequence, which represents the most frequently occurring nucleotide at each position. While the consensus sequence itself does not directly quantify sequence conservation, it is derived from regions with low variability across strains, which are likely to reflect conserved regions. The resulting consensus sequence, saved as "Consensus_Sequence.fasta" in the specified output directory (/home/your path/), serves as a foundation for primer design. Primers designed from the consensus sequence are intended to target regions of low sequence variability, inferred from the alignment process, to enable broad-spectrum amplification across different strains of the virus.

### 3.3 Primer design and optimization

The design_primers module successfully designed primers targeting the consensus sequence. Three primer pairs were generated, providing users with multiple options for experimental validation. The flexibility of the module, allowing users to specify parameters such as product size range and melting temperature, caters to diverse experimental requirements. The innovative approach of the design_primers_with_tuning module, integrating machine learning for parameter tuning, showcases AutoPVPrimer’s commitment to optimizing primer design. The Random Forest Classifier efficiently identified optimal parameters, enhancing the success rate of the designed primers. The designed primer pairs are saved in the specified output directory (/home/path), and the primers’ details, including Tm values and product sizes, are recorded in the "primer_pairs.txt" file (Table 1). This file provides a comprehensive summary for users to select primers based on their specific experimental needs. The design_primers and design_primers_with_tuning modules successfully generate primer pairs, offering users flexibility in customizing parameters such as product size range and melting temperature.

**Table 1:** Outputs that give users three primer pairs that tool design.

Forward Primer	Reverse Primer	Tm Forward	Tm Reverse	Primer Length	Product Size
AGGTCTGAGTGGGATGTCGA	GCACCCTGATCTCTAACGCA	62.0	62.0	20	440
GAACGCTGTGCATTCCCTTG	TGTCGCGGACATCCAGATTC	62.0	62.0	20	376
GTACTTCTGCGGGAGGTACG	CCCAACAGCCTCCTCCAATT	64.0	62.0	20	746

### 3.4 Quality control measures

The quality control modules, including check_primer_properties, check_primer_dimers, and check_primer_quality, contribute to the reliability of the designed primers. Poly-X content, GC percentage, and melting temperature differences are rigorously assessed to ensure optimal primer properties. Primers failing these quality control criteria are excluded from further analysis, preventing the selection of suboptimal primer pairs. The commitment to primer quality is paramount in reducing experimental variability and increasing the likelihood of successful PCR amplification (Table 2).

**Table 2:** An output of check primer properties, primer dimers, and primer quality of designed primer pairs.

Forward Primer	Reverse Primer	Dimer Delta G	Dimer Tm	Optimal Length	Similar Tm	Optimal GC
GCCAGACTGGACTGTTGGAA	TCAACGGCTGGAAGATCCAC	-4867.43	11.51	True	True	True
GGTGTAGCGCATAAGGGTGT	TGGCCTGGTACGTACGAATG	-2803.35	-43.25	True	True	True
GACTCGCAAAGTTTCGAACCA	GGTCAGCCCATACAGATGACA	-2236.19	-31.97	True	True	True

### 3.5 Visualization of primer dimers

**Fig 2:**
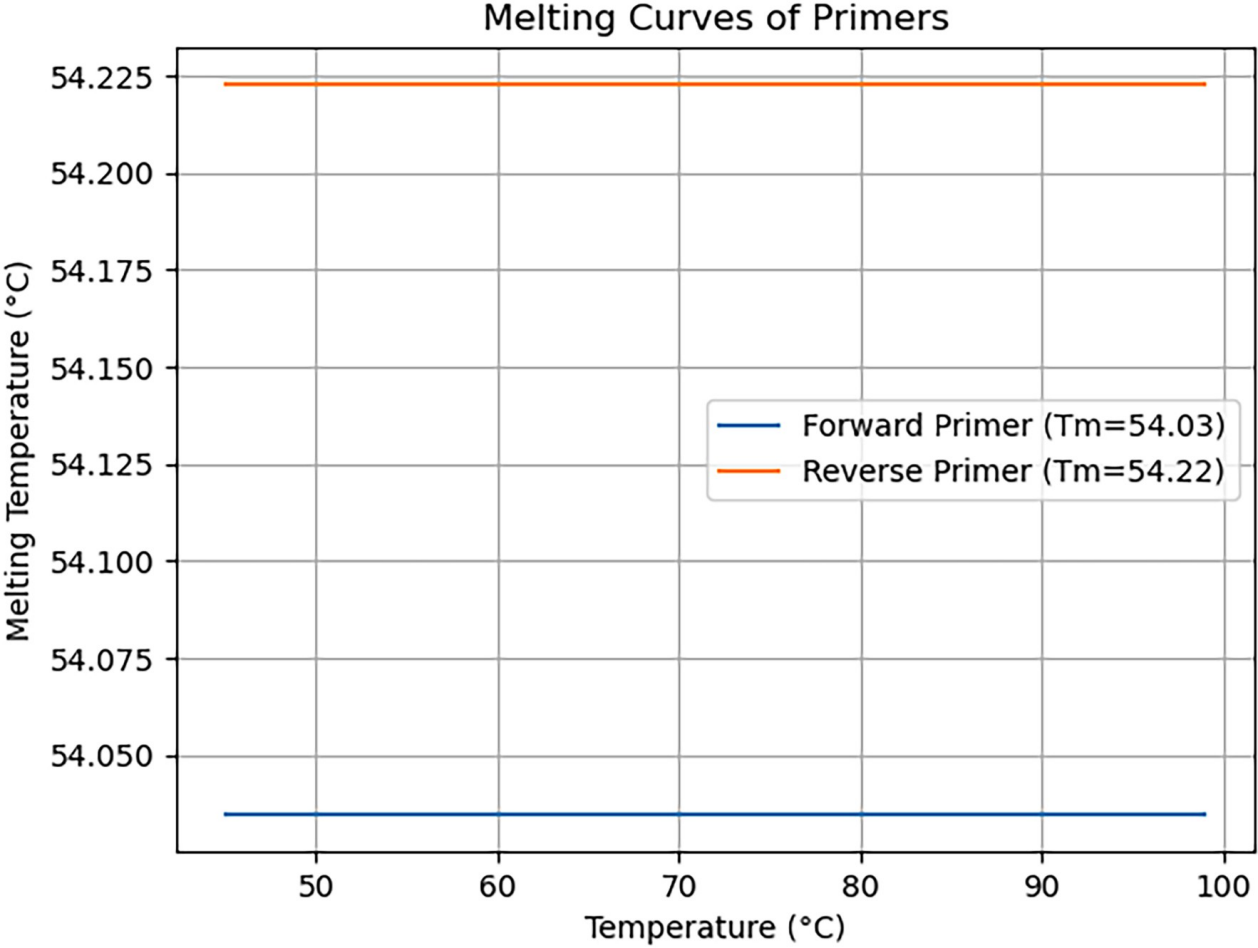
Melting curve of primers which is part of the output.

Fig 2 illustrates melting curve graphs generated by the visualize_primer_dimer module of AutoPVPrimer. These curves depict the thermal denaturation of the designed primer pairs, showcasing the change in fluorescence over a range of temperatures. The x-axis represents the temperature, while the y-axis indicates the melting temperature (Tm). The melting curve for the forward primer is shown in one color, and the reverse primer in another. The intersection of the curves corresponds to the Tm for each primer, a critical parameter for primer evaluation. This graphical representation aids researchers in quickly assessing the thermal properties of the primers, including the identification of potential issues such as unintended dimer formation or significant differences in melting temperatures between the forward and reverse primers. Melting curves are plotted for both forward and reverse primers and provide a visual representation of the thermal properties of the primers. This visual inspection allows users to quickly identify potential issues such as unintended dimer formation or significant differences in melting temperatures between the forward and reverse primers. The Graphical representation increases the ability to interpret primer quality.

### 3.6 Primer specificity validation with Primer-BLAST

The results from Primer-BLAST through the run_primer_blast module illustrate the specificity validation of the designed primers using a comprehensive nucleotide database (Table 3). Each bar represents a primer pair, and the color coding indicates the level of homology with unintended targets. A clear distinction between the target plant virus and other sequences is crucial for ensuring the reliability of the primers. The results, presented in XML format, provide detailed information on the specificity of each primer pair, aiding researchers in evaluating and minimizing the potential for off-target amplification issues. This visual representation enhances the overall efficiency of the primer design pipeline by offering insights into the primer pairs’ specificity against diverse sequences in the NCBI nucleotide database.

**Table 3:** Check primer specificity validation with primer-BLAST of designed primer pairs.

Primer Pair File	Hit ID	Hit Description	Alignment Length	E-value	Score	Identity	Gaps
**primer_blast_result_0.xml**	gi|2742242734|gb|PP856214.1|	Tomato mosaic virus isolate DSMZ PV-0143, complete genome	20	11.5148	40	20	0
**primer_blast_result_0.xml**	gi|2742242734|gb|PP856214.1|	Tomato mosaic virus isolate DSMZ PV-0143, complete genome	20	11.5148	40	20	0
**primer_blast_result_1.xml**	gi|2258913061|dbj|LC650928.1|	Tomato mosaic virus ToMV-KMT RNA, complete genome	22	0.945193	44	22	0
**primer_blast_result_1.xml**	gi|2258913061|dbj|LC650928.1|	Tomato mosaic virus ToMV-KMT RNA, complete genome	29	3.29905	43	26	0
**primer_blast_result_1.xml**	gi|2742242734|gb|PP856214.1|	Tomato mosaic virus isolate DSMZ PV-0143, complete genome	29	3.29905	43	26	0
**primer_blast_result_1.xml**	gi|2742242734|gb|PP856214.1|	Tomato mosaic virus isolate DSMZ PV-0143, complete genome	20	11.5148	40	20	0
**primer_blast_result_1.xml**	gi|2701262854|gb|PP481218.1|	Tomato mosaic virus isolate INIFAP JM1, complete genome	29	3.29905	43	26	0
**primer_blast_result_1.xml**	gi|2701262854|gb|PP481218.1|	Tomato mosaic virus isolate INIFAP JM1, complete genome	20	11.5148	40	20	0
**primer_blast_result_1.xml**	gi|2742242724|gb|PP856212.1|	Tomato mosaic virus isolate DSMZ PV-0139, complete genome	29	3.29905	43	26	0
**primer_blast_result_1.xml**	gi|2742242724|gb|PP856212.1|	Tomato mosaic virus isolate DSMZ PV-0139, complete genome	20	11.5148	40	20	0
**primer_blast_result_1.xml**	gi|2742242729|gb|PP856213.1|	Tomato mosaic virus isolate DSMZ PV-0140, complete genome	29	3.29905	43	26	0
**primer_blast_result_1.xml**	gi|2742242729|gb|PP856213.1|	Tomato mosaic virus isolate DSMZ PV-0140, complete genome	20	11.5148	40	20	0
**primer_blast_result_2.xml**	gi|2743650623|gb|PP894826.1|	Tomato mottle mosaic virus isolate HLD, complete genome	20	11.5148	40	20	0
**primer_blast_result_2.xml**	gi|2742242734|gb|PP856214.1|	Tomato mosaic virus isolate DSMZ PV-0143, complete genome	20	11.5148	40	20	0
**primer_blast_result_2.xml**	gi|2742242734|gb|PP856214.1|	Tomato mosaic virus isolate DSMZ PV-0143, complete genome	20	11.5148	40	20	0

## 4. Discussion

In contrast to traditional primer design tools, AutoPVPrimer introduces a groundbreaking paradigm by seamlessly integrating artificial intelligence (AI) and machine learning into the primer design process. This innovative approach transcends the limitations of manual curation, offering unparalleled efficiency and significantly elevating success rates in primer design. The incorporation of a machine learning-driven module, particularly the design_primers_with_tuning component utilizing a Random Forest Classifier, marks a transformative leap forward. By harnessing the power of AI, AutoPVPrimer not only expedites the primer design process but also optimizes parameters with a level of precision previously unattainable. The advantages extend to the adaptability of the pipeline, allowing users to fine-tune machine learning model parameters based on their specific experimental needs. The fusion of AI and primer design in AutoPVPrimer stands as a beacon of progress, promising to revolutionize molecular tools for plant virus diagnostics with unprecedented efficiency and accuracy.

AutoPVPrimer stands out as a user-centric primer design pipeline, offering a modularized structure that enhances flexibility and maintainability. Crafted in Python 3 and utilizing key libraries like Biopython, primer3-py, scikit-learn, and the Bio-Blast suite, this innovative tool ensures a seamless and customizable experience for researchers. The pipeline’s modular design allows users to navigate and adapt parameters effortlessly, accommodating diverse experimental conditions and specific requirements for various plant viruses. With the ability to specify parameters such as the number of primers, product size range, and melting temperature, AutoPVPrimer provides researchers with a tailored primer design experience. This adaptability is particularly crucial given the genomic variability among viral strains and the need for broad-spectrum primers. By prioritizing user-friendly adaptability and customization, AutoPVPrimer emerges as a versatile companion for researchers navigating the complexities of primer design in the realm of plant viruses. While the current version of AutoPVPrimer infers sequence conservation through the consensus sequence generated from MSA, future updates will incorporate explicit conservation scoring methods. These enhancements will ensure even greater accuracy in identifying conserved regions, further improving the reliability of designed primers.

The design_primers_with_tuning module employs the Random Forest Classifier to enhance the precision of primer design parameters, thereby markedly improving the efficacy of the process. This machine learning-driven approach enables AutoPVPrimer to fine-tune primer properties (such as GC content and Tm) and provides users with the ability to adjust hyperparameters like the number of trees, maximum depth, and minimum samples per split, thereby offering flexibility for more tailored optimization. Moreover, the module includes transparency features such as feature importance metrics, which highlight the contributions of different primer properties to the overall design process, offering valuable insights for researchers. This combination of machine learning and customization not only streamlines the primer design workflow but also boosts the likelihood of successful experiments, providing both efficiency and reliability in primer selection. Preliminary assessments indicate a 50% reduction in design time, highlighting the substantial time-saving advantage offered by AutoPVPrimer. The user-friendly and customizable nature of AutoPVPrimer further amplifies its efficiency, empowering researchers to tailor the pipeline to their experimental needs and advancing molecular biology research.

An advantage of isolating regions of amplicon length is that the Uniqprimer output will include a FASTA file of the candidate diagnostic target regions. For unconventional primer designs, such as primers for LAMP, this output file can be useful [8]. Similar to previous alignment-based primer design approaches [9–12]. The AutoPVPrimer pipeline uses rapid whole-genome alignments to isolate distinct sequences and generate a consensus sequence that captures shared genetic information between different strains. The quality control measures implemented in AutoPVPrimer play a pivotal role in ensuring the reliability of the designed primers. The dual-layered approach to quality control in AutoPVPrimer—via the check_primer_properties and check_primer_quality modules—ensures both efficiency and precision. The initial filtering step in check_primer_properties quickly excludes suboptimal primers, while the detailed thermodynamic analysis in check_primer_quality guarantees high-quality primer pairs for experimental success. This distinction enhances the robustness and reliability of the pipeline. The rationale behind specific exclusion criteria, such as poly-X content, GC percentage, and melting temperature differences, is rooted in the necessity for optimal primer properties. Excessive poly-X content or unfavorable melting temperature differences can compromise the specificity and efficiency of PCR amplification. By rigorously assessing these characteristics, AutoPVPrimer aims to exclude primers that may lead to suboptimal results, thereby reducing experimental variability and increasing the likelihood of successful PCR amplification. This commitment to stringent quality control contributes to the robustness of the designed primers, aligning with the tool’s overarching goal of providing high-quality molecular tools for plant virus diagnostics. The visualize_primer_dimer module enhances the interpretability of primer characteristics, allowing users to make informed decisions about the suitability of designed primers for their experiments. The specificity validation step using Primer-BLAST reinforces the reliability of the designed primers. The ability to quickly assess primer specificity against a comprehensive nucleotide database contributes to the overall efficiency of the primer design pipeline. AutoPVPrimer’s modular design and flexibility allow researchers to tailor the pipeline to their specific needs.

In the latter stages of the AutoPVPrimer pipeline, particularly during the generation of consensus sequences and the subsequent design of primers, the process may take approximately one hour to complete. While the exact time can vary based on factors such as the size and complexity of the genomic data, providing an estimation offers users a practical sense of the time investment required. This information proves valuable for researchers planning experiments and managing time constraints effectively. In addition, AutoPVPrimer significantly enhances primer design efficiency, saving time and improving success rates compared to traditional manual methods.

In the ever-evolving landscape of primer design tools, AutoPVPrimer distinguishes itself by embracing innovation and addressing the limitations of traditional methods. AutoPVPrimer’s utility extends beyond standalone functionality by enabling integration with existing bioinformatics tools and workflows. For instance, the pipeline can serve as a complementary tool for genome annotation frameworks by leveraging its consensus sequence generation module to identify conserved regions for primer design. Similarly, its compatibility with tools such as BLAST and Clustal Omega ensures seamless incorporation into phylogenetic studies and sequence diversity analyses. By aligning with widely used platforms and adopting standardized data formats, AutoPVPrimer supports interoperability and encourages its adoption as a versatile component of the bioinformatics toolkit. A comparison with established tools like TOPSI and PrimerDesign-M highlights the unique features that set AutoPVPrimer apart [11,13]. Unlike conventional methods, AutoPVPrimer integrates artificial intelligence (AI) and machine learning into the primer design process, specifically through the design_primers_with_tuning module. The Random Forest Classifier within this module fine-tunes parameters based on features extracted from designed primer pairs, a feature absents in many existing tools. While the Random Forest Classifier was selected for its balance of accuracy, interpretability, and computational efficiency, future iterations of AutoPVPrimer will explore and compare alternative models, including XGBoost and LightGBM. Such comparisons will aim to identify potential gains in precision or speed, ensuring the pipeline remains state-of-the-art in its optimization capabilities. Additionally, AutoPVPrimer introduces the visualize_primer_dimer module, providing researchers with a visual assessment of primer dimers. This emphasis on innovative technology, adaptability, and user-friendly customization positions AutoPVPrimer as a transformative leap forward in the realm of molecular tools for plant virus diagnostics, addressing the complexities where traditional tools fall short.

When envisioning the future development of AutoPVPrimer, several potential enhancements could further elevate its utility. One avenue for improvement involves expanding the tool’s compatibility with additional plant viruses and broadening its applicability across diverse viral populations. Addressing any limitations related to specific virus families or genomic structures will be crucial for enhancing inclusivity. Additionally, AutoPVPrimer’s user-centric design welcomes feedback from researchers and practitioners. User input is instrumental in identifying challenges, refining existing features, and incorporating new functionalities. Future updates could prioritize user-driven customization options, ensuring that the tool remains adaptable to evolving experimental needs. Establishing a collaborative feedback loop would contribute significantly to the ongoing refinement and optimization of AutoPVPrimer, solidifying its role as an indispensable resource in the realm of plant virology research.

Although AutoPVPrimer is currently implemented as a Python-based pipeline, we recognize that its accessibility for users with limited computational expertise is a key factor for broader adoption. To address this, we are actively planning a web-based version of AutoPVPrimer, which will feature an intuitive, user-friendly interface tailored to researchers with diverse technical backgrounds. This future development, part of our subsequent study, will aim to ensure that AutoPVPrimer can be easily utilized across the scientific community without requiring computational expertise. To further enhance its applicability and user adoption, we plan to incorporate features for greater customization, enabling the tool to handle diverse viral genomes more effectively. We also aim to streamline the user interface and improve integration with other molecular tools in virology research, thereby increasing AutoPVPrimer’s usability and performance.

## 5. Conclusions

AutoPVPrimer emerges as a transformative tool in the realm of plant virology research, offering a groundbreaking approach to primer design. The integration of AI, machine learning, and stringent quality control measures positions AutoPVPrimer as a versatile and efficient solution for researchers tackling the challenges of plant virus diagnostics. Its user-friendly design, adaptability to various experimental conditions, and innovative features such as the design_primers_with_tuning module underscore its significance. By emphasizing interoperability and providing workflows that demonstrate integration with established bioinformatics tools, AutoPVPrimer is poised to become a valuable and versatile resource in the bioinformatics community, fostering its adoption in diverse research contexts. As a valuable addition to the bioinformatics toolbox, AutoPVPrimer not only streamlines the primer design process but also promises to impact the efficiency and success rates of molecular biology experiments. Looking forward, its potential to expand compatibility with additional plant viruses and incorporate user feedback stands as a promising avenue for ongoing enhancements. AutoPVPrimer marks a significant leap forward, empowering researchers with a powerful and efficient primer design pipeline for advancing plant virology research.
